# Gluteal Muscle Atrophy and Increased Intramuscular Lipid Concentration Are Not Mitigated by Daily Artificial Gravity Following 60-Day Head-Down Tilt Bed Rest

**DOI:** 10.3389/fphys.2021.745811

**Published:** 2021-11-11

**Authors:** Vienna Tran, Enrico De Martino, Julie Hides, Gordon Cable, James M. Elliott, Mark Hoggarth, Jochen Zange, Kirsty Lindsay, Dorothée Debuse, Andrew Winnard, David Beard, Jonathan A. Cook, Sauro E. Salomoni, Tobias Weber, Jonathan Scott, Paul W. Hodges, Nick Caplan

**Affiliations:** ^1^Adelaide Medical School, The University of Adelaide, Adelaide, SA, Australia; ^2^Aerospace Medicine and Rehabilitation Laboratory, Faculty of Health and Life Sciences, Northumbria University, Newcastle upon Tyne, United Kingdom; ^3^School of Health Sciences and Social Work, Griffith University, Brisbane, QLD, Australia; ^4^School of Medicine, University of Tasmania, Hobart, TAS, Australia; ^5^Department of Physical Therapy and Human Movement Sciences, Feinberg School of Medicine, Northwestern University, Chicago, IL, United States; ^6^Faculty of Medicine and Health, The Kolling Research Institute Sydney, Northern Sydney Local Health District, The University of Sydney, Sydney, NSW, Australia; ^7^Department of Biomedical Engineering, McCormick School of Engineering, Northwestern University, Evanston, IL, United States; ^8^Institute of Aerospace Medicine, German Aerospace Center (DLR), Cologne, Germany; ^9^Nuffield Department of Orthopaedics, Rheumatology and Musculoskeletal Sciences, NIHR Oxford Biomedical Research Centre, University of Oxford, Oxford, United Kingdom; ^10^Nuffield Department of Orthopaedics, Rheumatology and Musculoskeletal Sciences, Centre for Statistics in Medicine, University of Oxford, Oxford, United Kingdom; ^11^NHMRC Centre for Clinical Research Excellence in Spinal Pain, Injury and Health, School of Health and Rehabilitation Sciences, The University of Queensland, Brisbane, QLD, Australia; ^12^Space Medicine Team (HRE-OM), European Astronaut Centre, Cologne, Germany; ^13^KBR GmbH, Cologne, Germany

**Keywords:** AGBRESA bed rest, Dixon sequence, short-arm centrifugation, intramuscular fatty infiltration, gluteal muscles, muscle atrophy

## Abstract

Exposure to spaceflight and head-down tilt (HDT) bed rest leads to decreases in the mass of the gluteal muscle. Preliminary results have suggested that interventions, such as artificial gravity (AG), can partially mitigate some of the physiological adaptations induced by HDT bed rest. However, its effect on the gluteal muscles is currently unknown. This study investigated the effects of daily AG on the gluteal muscles during 60-day HDT bed rest. Twenty-four healthy individuals participated in the study: eight received 30 min of continuous AG; eight received 6 × 5 min of AG, interspersed with rest periods; eight belonged to a control group. T1-weighted Dixon magnetic resonance imaging of the hip region was conducted at baseline and day 59 of HDT bed rest to establish changes in volumes and intramuscular lipid concentration (ILC). Results showed that, across groups, muscle volumes decreased by 9.2% for gluteus maximus (GMAX), 8.0% for gluteus medius (GMED), and 10.5% for gluteus minimus after 59-day HDT bed rest (all *p* < 0.005). The ILC increased by 1.3% for GMAX and 0.5% for GMED (both *p* < 0.05). Neither of the AG protocols mitigated deconditioning of the gluteal muscles. Whereas all gluteal muscles atrophied, the ratio of lipids to intramuscular water increased only in GMAX and GMED muscles. These changes could impair the function of the hip joint and increased the risk of falls. The deconditioning of the gluteal muscles in space may negatively impact the hip joint stability of astronauts when reexpose to terrestrial gravity.

## Introduction

Because the musculoskeletal system has evolved within the Earth’s gravitational field, humans possess a complex weight-bearing system that facilitates bipedal standing balance, gait, and protection against joint injury during functional tasks (Richardson, 2002). When weight-bearing is severely limited, such as during space missions, the reduced mechanical stress to skeletal muscle leads to rapid neuromuscular adaptations, defined as muscle deconditioning (Parry and Puthucheary, 2015). As there are few astronauts to study, an alternative is ground-based analog studies, such as strict head-down tilt (HDT) bed rest, to understand the effect of prolonged axial (gravitational) unloading upon the weight-bearing muscles. Results from healthy individuals placed in bed for more than 30 days have provided crucial insights into the negative consequences of inactivity on the muscular system (Narici et al., 2020). HDT bed rest studies have been used as a model to simulate the effects of microgravity on the human body since the 6° head-down position on Earth provokes similar adaptations described in astronauts after spaceflight (Pavy-Le Traon et al., 2007). Investigation of the fundamental mechanisms underlying the effect of prolonged decreased axial loading on weight-bearing muscles is crucial to inform the design of optimal interventions aimed at muscle reconditioning and rehabilitation.

Muscle atrophy is one of the indicators of musculoskeletal deconditioning (Phillips et al., 2009). Several studies of healthy people following exposure to spaceflight and bed rest have shown larger reductions in muscle strength than muscle volume measures derived from images, such as magnetic resonance imaging (MRI) (Zange et al., 1997; Kawakami et al., 2001; Trappe et al., 2009), which suggests that neuromuscular adaptations (Dudley et al., 1992; Berg et al., 1997) and changes in properties of muscle tissue are likely to contribute to the impaired muscle function (Trappe et al., 2004, 2009). Another indicator of deconditioning which can be determined from MRI is intramuscular lipid concentration (ILC). Changes in ILC after reduced physical activity have been suggested to alter mechanical properties of muscle that may contribute to loss of muscle function (Manini et al., 2007). In healthy adults, extreme physical inactivity attenuates fatty acid oxidation in skeletal muscles, which creates an environment conducive to the accumulation of adipose tissue (Manini et al., 2007). In this process, the precursors to skeletal muscle cells, satellite cells, differentiate into adipocytes rather than into mature skeletal muscle cells (Sanna et al., 2009). As a result, healthy adults with lower physical activity have a greater ILC in their weight-bearing muscles than individuals with higher levels of physical activity, regardless of age (Crawford et al., 2015).

Magnetic resonance imaging is used to quantify muscle architecture and properties of muscle tissue and has been used to evaluate changes in the size of several weight-bearing muscles, including calf muscles (Trappe et al., 2009), quadriceps (Belavý et al., 2009), lumbar (Bailey et al., 2018; Hides et al., 2020), and neck muscles (McNamara et al., 2019) after prolonged bed rest and space missions. Few studies have documented changes in the gluteal muscles (Miokovic et al., 2011). ILC is best evaluated with multi-echo Dixon sequences, which display adipose tissue separately from muscle and collect data on fat and water, both in-phase and out-of-phase (Dixon, 1984; Elliott et al., 2013). These data can be combined to create a fat and water image to reliably quantify the content of each (Elliott et al., 2013). The reliability of this technique is comparable with muscle biopsy ILC (Smith et al., 2014). It is currently unknown whether a prolonged period of reduced axial loading increases the ILC in the gluteal muscles.

The gluteal muscle group includes the gluteus maximus (GMAX), gluteus medius (GMED), and gluteus minimus (GMIN) muscle. The GMED and GMIN muscles are critical in weight-bearing to control the pelvis and the femoral head during single-leg stance and gait (Gottschalk et al., 1989; Semciw et al., 2013, 2014), and the GMAX muscle accelerates the body upward and forward from a position of hip flexion, such as when standing up from a seated position or climbing steps (Neumann, 2010). The difference in the function of individual gluteal muscles suggests that a detailed analysis of each muscle is necessary to better understand any potential changes induced by prolonged disuse. Furthermore, the pathology that affects the strength, motor control, or extensibility of the gluteal muscles significantly disrupts many daily movements involving functional and recreational activities, such as standing, walking, and stepping (Retchford et al., 2013). Although HDT bed rest has been shown to induce atrophy of the gluteal muscles, with greater atrophy of GMIN (∼11%) than the GMED (∼4%) and GMAX (∼7%) (Miokovic et al., 2011), no previous studies have investigated ILC in the gluteal muscles following HDT bed rest. Whether ILC accumulates differently in the three muscles is also not clear.

Artificial gravity (AG) has been shown to positively affect the human body (Kotovskaya, 2010; Clément G. R. et al., 2015), and, for this reason, it has been brought to the attention of the major space agencies as a possible countermeasure against human body deconditioning (Clément, 2017). AG can be generated by utilizing a short-arm centrifuge (3.8-m radius) to produce a head-to-foot force on the body while in a supine lying position. At specific speeds, this head-to-foot force is similar to the force of gravity on the body when in a standing position on Earth (Clément G. R. et al., 2015). Preliminary results from HDT bed rest studies have suggested that daily exposure to AG can mitigate deconditioning in the cardiovascular and neurovestibular systems (Stenger et al., 2012; Linnarsson et al., 2015), but the effects of AG on the gluteal muscles have not been investigated. Resistive exercise training in the supine lying position has been shown to mitigate gluteal muscle atrophy during 60-day HDT bed rest (Miokovic et al., 2011). Thus, daily AG may provide a similar protective mechanical stimulation on the gluteal muscles due to the axial head-to-foot force created by the centrifugation. Furthermore, whether the centrifugation is delivered in an intermittent or continuous protocol may also influence its tolerance and effectiveness, as intermittent AG has been shown to be marginally more tolerable in terms of cardiovascular loading and motion sickness (Frett et al., 2020). Intermittent AG has also mitigated orthostatic intolerance more effectively than continuous AG in a 5-day HDT bed rest study (Clément G. et al., 2015; De Martino et al., 2021b).

The primary aim of this study was to investigate whether exposure to 60-day HDT bed rest induced atrophy and increased ILC in the individual gluteal muscles. The second aim was to examine whether two daily AG protocols, intermittent and continuous, could mitigate any effects of 60-day HDT bed rest on the gluteal muscles. As AG is associated with a large acceleration gradient along the body axis, it was hypothesized that a mechanical compressive force on the gluteal muscles would stimulate the muscle cells, mitigating muscle deconditioning.

## Materials and Methods

### Participants and Group Characteristics

This study was part of the NASA/ESA/DLR Artificial Gravity Bed Rest study (AGBRESA), undertaken at the “:envihab” facility (German Aerospace Center) in Cologne (Koch and Gerzer, 2008), Germany, from March to December 2019. Participants attended the facility for baseline data collection (BDC) 14 days before commencing 60 days of strict 6° HDT bed rest. Originally, the study was planned with an equal number of male and female participants. However, the final cohort comprised 8 females and 16 males due to several drop-outs. Research studies examining multiple systems were conducted simultaneously in the AGBRESA study (Frett et al., 2020). The sample size of 24 was selected based on previous HDT bed rest studies demonstrating the protective effects of continuous and intermittent AG for 30 min on orthostatic tolerance (Linnarsson et al., 2015) and bone resorption (Rittweger et al., 2015). The study conformed with the “*International Guidelines for Standardization of Bed Rest Studies in the Spaceflight Context*” (Sundblad et al., 2014). These guidelines describe all specific aspects of the study design, including study durations, participant position, management, nomenclature, subject selection (inclusion and exclusion criteria), rules for and monitoring of subjects, physiotherapy, medical care and psychological support, ethics, definition and monitoring of nutritional intake, and handling of data and biological samples (Sundblad et al., 2014). This document was agreed upon among an international group of scientists and representatives of the space agencies to ensure control, quality, reliability, and repeatability of the HDT bed rest studies (Sundblad et al., 2016).

Participants were pain-free at BDC testing, and none reported a history of chronic or acute musculoskeletal disorders or other medical disorders that would affect the measures collected in the study. Each participant was randomly allocated to one of three intervention groups: a control (CTRL) group that was not exposed to AG, a group that underwent 30 min of continuous centrifugation (cAG) daily, or a group that underwent six sets of 5 min centrifugation (iAG) daily with 3 min of rest between each set.

All participants completed the 60 days of HDT bed rest. They performed all activities, including hygiene, in the supine position and were discouraged from moving unnecessarily. They were permitted to lie supine or on their side but were instructed to have at least one shoulder touching the bed at all times. Caloric intake was balanced with the measured individual metabolic energy consumption to maintain body mass throughout bed rest. Water intake was restricted to 50 mL/kg of body mass. Smoking, alcohol, and caffeinated drinks were not permitted. Participants followed a day-night cycle of 7 a.m. awakening and “lights-out” at 11 p.m.

The Ethics Committee of the Northern Rhine Medical Association approved this study (Düsseldorf, Germany, Application No. 2018143). The study was registered in the German Clinical Trial Register (DRKS) under No. DKRS00015677. Written informed consent was obtained before study commencement.

### Artificial Gravity

All centrifugation was performed using a short-arm human centrifuge of 3.8 m in radius (Koch and Gerzer, 2008), in accordance with the “International Roadmap for Artificial Gravity Research” (Clément, 2017). The rotation speed was 0.5 Hz to produce approximately 1 Gz at the heart level and 2 Gz at the feet. During the 60-day HDT bed rest, participants in the cAG and iAG groups were transferred on a 6° HDT gurney to the centrifuge facility. They were asked to roll over to the centrifuge without using their leg muscles, and they were placed on the centrifuge arm at 6° HDT. The participants were supine, with their feet directed outward. Restraints in the lower limbs, upper limbs, and trunk were used, but participants were instructed to keep their body and head still throughout the centrifugation as much as possible. During centrifugation, the cardiorespiratory parameters of the participants were continuously monitored. The participants could perform anti-orthostatic maneuvers, such as heel raises and shallow knee bends, to maintain blood circulation while spinning (Gardner et al., 1983), but were otherwise instructed to remain still. More details about the protocol are reported elsewhere (see Frett et al., 2020).

### Magnetic Resonance Imaging Procedure

Magnetic resonance imaging scans were conducted with the participants comfortably positioned in supine lying with their knees and hips supported in slight flexion by a pillow, using a 3 Tesla Magnetom Vision system (Siemens, Erlangen, Germany). Scans were performed in the transverse plane to image the gluteal muscles 2 days before the HDT bed rest period for BDC and again on the 59th day of HDT bed rest (HDT59). For each participant at each time point, 128 transverse images were acquired, covering the region from the T11 vertebral level to the inferior-most portion of the GMAX (T1 weighted 2-point Dixon sequence, slice thickness = 4 mm; distance factor = 20%, TR = 7.02 ms, TE1 = 2.46 ms, TE2 = 3.69 ms, flip angle = 5 deg; field of view = 400 mm × 400 mm at 1.0 mm × 1.0 mm pixel size). Images were obtained with the fat and water protons in-phase and out of phase, then fat (F) images and water images (W) were reconstructed. Measurements of the gluteal muscles on the MRIs were conducted after the study. MRIs were assigned a random code to ensure blinding of the measurer to time points and participant groups. Other imaging data from this study pertaining to the lumbar spine region, including muscle volume and ILC, have been published elsewhere (De Martino et al., 2021a).

### Gluteal Muscle Volume and Intramuscular Lipid Concentration

**FIGURE 1:**
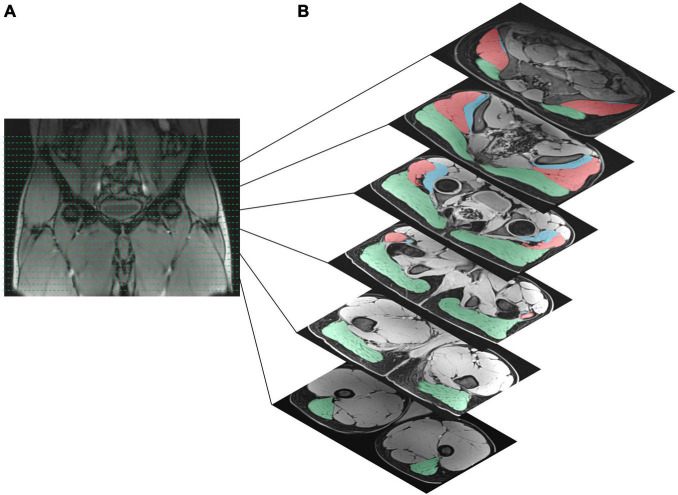
**(A)** Section of the pelvis on frontal images used for the analysis. **(B)** Characteristic location of gluteal muscles identified for the area measurement on axial images (slide thickness = 4 mm; slice gap = 20%). The muscle volume of the gluteus maximus muscle (green shaded area), gluteus medius muscle (red shaded area), and gluteus minimus muscle (blue shaded area) was calculated bilaterally from the origin to the insertion of each gluteal muscle.


Regions of interest (ROI) were manually segmented around the gluteal muscles (Figure 1), using a semi-automated MATLAB-based program (The MathWorks, Inc., Natick, MA, United States) (Mhuiris et al., 2016). The program automatically quantified the cross-sectional area and the ILC of each ROI, and the ILC was calculated as the ratio of pixel intensities from the F and W images, expressed as a percentage:

ILC=[F/(W+F)]× 100

For each gluteal muscle, the sum of cross-sectional areas was multiplied by the MRI slice’s thickness (0.4 cm) to obtain a muscle volume in cm^3^ for each participant. ILC measurements were averaged for each participant. For both volume and ILC, the left- and right-sided measurements were averaged before statistical analysis because both sides were assumed to be affected equally.

### Statistical Analysis

Statistical analysis was performed in Statistical Package for Social Sciences (SPSS; Version 25, IBM, Chicago, IL, United States). All results were presented as means with standard deviation (SD). Statistical significance was set at the (two-sided) 0.05 level. First, outcomes were assessed for normality using the Shapiro–Wilk test and visual inspection. A one-way analysis of variance (ANOVA) was used to test for between-group differences in participant and group characteristics at BDC. These characteristics included age, body mass, height, muscle volume, and ILC.

To examine the effect of the different AG interventions compared with the control group after bed rest, muscle volume and ILC (dependent variables) were analyzed using a three-way mixed-model ANOVA with *Group* (CRTL, cAG, and iAG – between-group factor), *Sex* (Female and Male – between-group factor), and *Time* (BDC and HDT59 – within-subject factor). Interactions between the factors were included in the model. Partial eta-squared (η^2^_partial_) was calculated since it reflects the proportion of variance in each effect or interaction and the error that is accounted for by that effect (Lakens, 2013).

Since the muscle volume and ILC in the gluteal muscles were different at BDC, the relative delta (Δ) of muscle volume and ILC was calculated (Δ = 100 × (HDT59 − BDC)/BDC), and a three-way mixed-model ANOVA was used between *Groups* (CRTL, cAG, and iAG; between-group factor), *Sex* (Female and Male – between-group factor), and *Muscles* (GMAX, GMED, and GMIN; within-subject factor). Interactions between the factors were included in the model, and η^2^_partial_ was calculated. The Greenhouse–Geisser approach was used to correct against violations of sphericity. Where appropriate, *post hoc* pairwise analyses were performed using Bonferroni corrected multiple comparisons, and corresponding confidence intervals (CIs) were generated. For missing data, no imputation was carried out.

## Results

### Participants and Group Characteristics

All participants attended their scheduled imaging sessions, and the MRIs were successfully collected from all participants. Because of chemical-shift artifacts, some images for a given muscle were excluded. A total of 9 muscles were excluded across five participants (GMAX = 1; GMED = 4; GMIN = 4), leaving 63 muscles with complete data included in the final analysis of volume and ILC (GMAX = 23; GMED = 20; GMIN = 20).

One operator conducted all MRI measurements, and the intra-rater reliability of the operator performing the transverse plane image measurements was evaluated before starting the analyses. The measurements were made twice with a minimum of 7 days apart (intraclass correlation coefficients: ICC_2,1_: range of 0.938–0.994; 95% CI: 0.905–0.999).

At BDC, one-way ANOVA revealed no *Group* differences in the sex distribution or age, height, body mass, body mass index, muscle volume, or muscle ILC of the participants (Table 1, all *p* > 0.05).

**TABLE 1:** Mean ± standard deviation of participant demographics and group characteristics at baseline data collection.

**Variable**	**CTRL**	**cAG**	**iAG**
Number of participants	8	8	8
Sex (F)	2	3	3
Age (years)	34 ± 8	32 ± 10	34 ± 11
Height (cm)	177 ± 7	173 ± 8	174 ± 11
Body mass (kg)	79 ± 13	72 ± 10	71 ± 5
Body mass index (kg/m^2^)	25 ± 3	24 ± 2	22 ± 2
**Muscle volume (cm^3^):**			
Gluteus maximus m.	3051 ± 956^∧^	3158 ± 892	2662 ± 750
Gluteus medius m.	551 ± 282^∧^	681 ± 177^∧^	504 ± 205^∧^
Gluteus minimus m.	265 ± 122^∧^	299 ± 047^∧^	232 ± 090^∧∧^
**Muscle ILC (%):**			
Gluteus maximus m.	13.8 ± 3.0^∧^	14.4 ± 4.9	16.2 ± 5.8
Gluteus medius m.	9.4 ± 2.9^∧^	8.8 ± 1.4^∧^	8.5 ± 1.4^∧^
Gluteus minimus m.	9.6 ± 3.6^∧^	9.8 ± 4.7^∧^	8.0 ± 1.6^∧∧^

*F, female; CRTL, control group; cAG, continuous artificial gravity group; iAG, intermittent artificial gravity group.*

*^∧^*n* = 7 due to imaging artifacts.*

*^∧∧^*n* = 6 due to imaging artifacts.*

### Gluteal Muscle Volume

**FIGURE 2:**
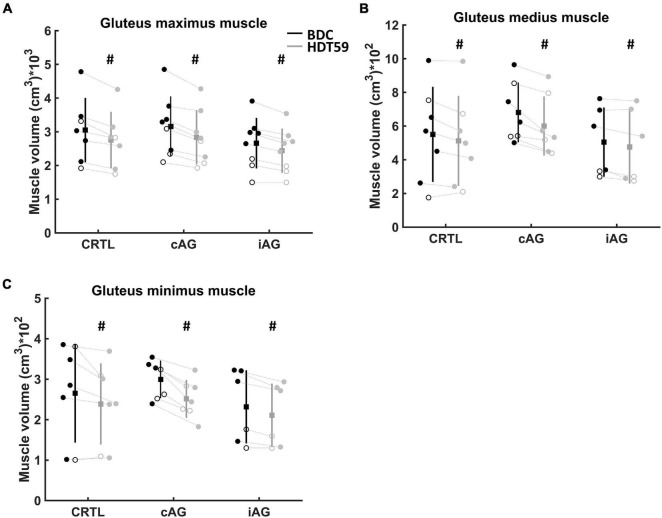
Changes in muscle volume over time. Circle represents female participants, and filled circle represents male participants. Group mean is a filled square, and the standard deviation is vertical lines. The black vertical line is baseline data collection (BDC), and gray vertical line is day 59 of head-down tilt (HDT59) bed rest. **(A)** Muscle volume of the gluteus maximus muscle. **(B)** Muscle volume of the gluteus medius muscle. **(C)** Muscle volume of the gluteus minimus muscle. Significantly lower muscle volume in the HDT59 compared with BDC (^#^*p* < 0.05).

An overview of the three groups’ average muscle volumes at BDC and HDT59 is reported in Table 2. The three-way mixed-model ANOVA revealed that all three gluteal muscles reduced the muscle volume after 59 days of HDT bed rest (main effect for *Time* – all *p* < 0.005; η^2^_partial_ > 0.5) (Figure 2). Across all groups, the gluteal muscle with the greatest decrease in volume was the GMIN (10.5 ± 8%), followed by the GMAX muscle (9.2 ± 3%) and GMED muscle (8.0 ± 10%). A main effect of *Sex* was only found in GMAX muscle (*p* = 0.009; η^2^_partial_ = 0.3), with males showing higher muscle volume (CI 95%: 268.2–1579.7). No differences between sexes were detected in GMED and GMIN muscles (both *p* > 0.05; η^2^_partial_ ≤ 0.1). No main effect of *Group* or interactions between factors were found (all *p* > 0.05; η^2^_partial_ ≤ 0.2) (Table 2).

**TABLE 2:** Mean ± standard deviation of gluteal muscle volumes (cm^3^) for the CTRL, cAG, and iAG groups.

	**Group**	**Time**		**Three-way mixed-model repeated-measures ANOVA**						
		**BDC**	**HDT59**	**Time**	**Group**	**Sex**	**Time-by-group**	**Time-by-sex**	**Group-by-sex**	**Time-by-group-by-sex**
GMAX muscle	CRTL	3051 ± 956	2756 ± 837	*F*_1,17_ = 71.1	*F*_2,17_ = 0.9	*F*_1,17_ = 8.8	*F*_2,17_ = 1.2	*F*_1,17_ = 0.8	*F*_2,17_ = 0.2	*F*_2,17_ = 0.7
	cAG	3158 ± 892	2835 ± 800	***p* < 0.001**	*p* = 0.428	***p* = 0.009**	*p* = 0.315	*p* = 0.376	*p* = 0.802	*p* = 0.493
	iAG	2662 ± 750	2439 ± 655	**η^2^_partial_ = 0.8**	η^2^_partial_ = 0.1	**η^2^_partial_ = 0.3**	η^2^_partial_ = 0.1	η^2^_partial_ = 0.0	η^2^_partial_ = 0.0	η^2^_partial_ = 0.1
GMED muscle	CRTL	551 ± 282	512 ± 267	*F*_1,17_ = 14.6	*F*_2,17_ = 1.1	*F*_1,17_ = 1.8	*F*_2,17_ = 1.7	*F*_1,17_ = 0.6	*F*_2,17_ = 0.4	*F*_2,17_ = 0.2
	cAG	681 ± 177	600 ± 175	***p* = 0.002**	*p* = 0.340	*p* = 0.198	*p* = 0.207	*p* = 0.440	*p* = 0.663	*p* = 0.834
	iAG	504 ± 205	476 ± 217	**η^2^_partial_ = 0.5**	η^2^_partial_ = 0.1	η^2^_partial_ = 0.1	η^2^_partial_ = 0.1	η^2^_partial_ = 0.1	η^2^_partial_ = 0.1	η^2^_partial_ = 0.0
GMIN muscle	CRTL	265 ± 122	210 ± 075	*F*_1,17_ = 30.8	*F*_2,17_ = 0.9	*F*_1,17_ = 1.8	*F*_2,17_ = 3.0	*F*_1,17_ = 1.5	*F*_2,17_ = 0.4	*F*_2,17_ = 1.0
	cAG	299 ± 047	251 ± 047	***p* < 0.001**	*p* = 0.420	*p* = 0.201	*p* = 0.078	*p* = 0.246	*p* = 0.660	*p* = 0.396
	iAG	232 ± 090	211 ± 078	**η^2^_partial_ = 0.7**	η^2^_partial_ = 0.1	η^2^_partial_ = 0.1	η^2^_partial_ = 0.2	η^2^_partial_ = 0.1	η^2^_partial_ = 0.1	η^2^_partial_ = 0.1

*BDC, baseline data collection; HDT59, day 59 of head-down tilt bed rest; CRTL, control group; cAG, continuous artificial gravity group; iAG, intermittent artificial gravity group. Boldface indicates *P* < 0.05.*

The three-way mixed-model ANOVA on relative Δ did not reveal a main effect of *Muscle*, *Group*, *Sex*, and interactions between factors (Table 3).

**TABLE 3:** Mean ± standard deviation of relative Δ of the gluteal muscle volumes for the CTRL, cAG, and iAG groups.

**Group**	**Muscle**			**Three-way mixed-model repeated-measures ANOVA**						
	**GMAX**	**GMED**	**GMIN**	**Muscle**	**Group**	**Sex**	**Muscle-by-group**	**Muscle-by-sex**	**Group-by-sex**	**Time-by-muscle-by-sex**
CRTL	−9.4 ± 3.1	−4.6 ± 13.3	−6.9 ± 10.4	*F*_2,26_ = 1.2	*F*_2,13_ = 1.8	*F*_1,13_ = 0.9	*F*_4,26_ = 1.6	*F*_2,26_ = 2.1	*F*_2,13_ = 0.1	*F*_4,26_ = 1.6
cAG	−10.4 ± 4.0	−12.1 ± 7.1	−16.2 ± 6.3	*p* = 0.325	*p* = 0.211	*p* = 0.351	*p* = 0.214	*p* = 0.136	*p* = 0.928	*p* = 0.196
iAG	−7.9 ± 3.3	−7.2 ± 5.7	−8.2 ± 4.3	η^2^_partial_ = 0.1	η^2^_partial_ = 0.2	η^2^_partial_ = 0.1	η^2^_partial_ = 0.2	η^2^_partial_ = 0.1	η^2^_partial_ = 0.0	η^2^_partial_ = 0.2

*CRTL, control group; cAG, continuous artificial gravity group; iAG, intermittent artificial gravity group.*

### Gluteal Muscle Intramuscular Lipid Concentration

**FIGURE 3:**
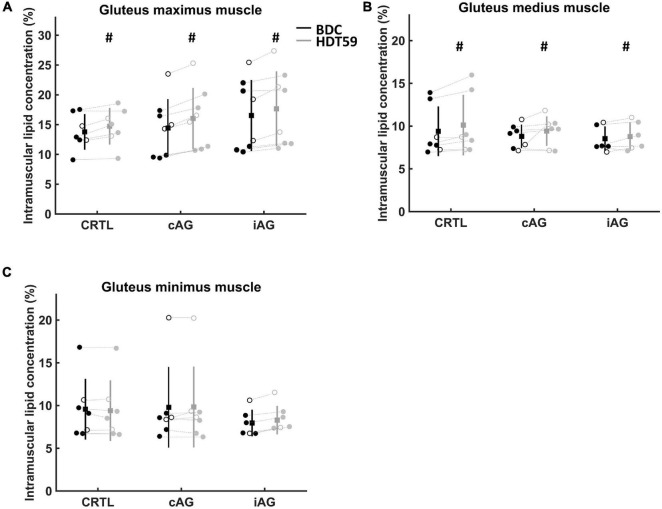
Changes in intramuscular lipid concentration over time. Circle represents female participants, and filled circle represents male participants. Group mean is a filled square, and the standard deviation is vertical lines. The black vertical line is baseline data collection (BDC), and gray vertical line is day 59 of head-down tilt (HDT59) bed rest. **(A)** Intramuscular lipid concentration into the gluteus maximus muscle. **(B)** Intramuscular lipid concentration into the gluteus medius muscle. **(C)** Intramuscular lipid concentration into the gluteus minimus muscle. Significantly higher ILC in the HDT59 compared with BDC (^#^*p* < 0.05). Intramuscular lipid concentration %-value refers to the whole H1 signal.

An overview of the average muscle ILCs for the three groups at BDC and HDT59 is reported in Table 4. The three-way mixed-model ANOVA revealed an increase in GMAX and GMED ILC after 59 days of HDT bed rest (main effect for *Time* – both *p* < 0.05; η^2^_partial_ > 0.3) (Figure 3). No change was detected in GMIN muscle (*p* = 0.25; η^2^_partial_ = 0.1). Across all groups, the absolute change of ILC from BDC was 1.5 ± 1.0% in the GMAX muscle and 0.5 ± 0.9% in the GMED muscle. No main effect of *Group*, *Sex*, and interactions between factors were found (all *p* > 0.05; η^2^_partial_ ≤ 0.2).

**TABLE 4:** Mean ± standard deviation of gluteal muscle intramuscular lipid concentrations (%) for the CTRL, cAG, and iAG groups.

	**Group**	**Time**		**Mixed-model repeated-measures ANOVA**						
		**BDC**	**HDT59**	**Time**	**Group**	**Sex**	**Time-by-group**	**Time-by-sex**	**Group-by-sex**	**Time-by-group-by-sex**
GMAX muscle	CRTL	13.8 ± 3.0	14.7 ± 3.0	*F*_1,17_ = 35.8	*F*_2,17_ = 0.5	*F*_1,17_ = 2.2	*F*_2,17_ = 1.0	*F*_1,17_ = 0.1	*F*_2,17_ = 0.5	*F*_2,17_ = 0.5
	cAG	14.4 ± 4.9	16.0 ± 5.2	***p* < 0.001**	*p* = 0.478	*p* = 0.156	*p* = 0.338	*p* = 0.818	*p* = 0.574	*p* = 0.631
	iAG	16.2 ± 5.8	17.7 ± 6.2	**η^2^_partial_ = 0.7**	η^2^_partial_ = 0.1	η^2^_partial_ = 0.1	η^2^_partial_ = 0.1	η^2^_partial_ = 0.0	η^2^_partial_ = 0.1	η^2^_partial_ = 0.1
GMED muscle	CRTL	9.4 ± 2.9	10.1 ± 3.6	*F*_1,17_ = 5.7	*F*_2,17_ = 0.1	*F*_1,17_ = 0.4	*F*_2,17_ = 0.3	*F*_1,17_ = 0.1	*F*_2,17_ = 0.7	*F*_2,17_ = 2.7
	cAG	8.8 ± 1.4	9.4 ± 1.7	***p* = 0.032**	*p* = 0.941	*p* = 0.548	*p* = 0.745	*p* = 0.940	*p* = 0.548	*p* = 0.102
	iAG	8.5 ± 1.4	8.8 ± 1.7	**η^2^_partial_ = 0.3**	η^2^_partial_ < 0.1	η^2^_partial_ = 0.0	η^2^_partial_ = 0.0	η^2^_partial_ = 0.0	η^2^_partial_ = 0.1	η^2^_partial_ = 0.2
GMIN muscle	CRTL	9.6 ± 3.6	9.4 ± 3.6	*F*_1,17_ = 1.4	*F*_2,17_ = 0.6	*F*_1,17_ = 0.3	*F*_2,17_ = 1.7	*F*_1,17_ = 3.2	*F*_2,17_ = 1.5	*F*_2,17_ = 0.1
	cAG	9.8 ± 4.7	9.8 ± 4.7	*p* = 0.251	*p* = 0.562	*p* = 0.618	*p* = 0.219	*p* = 0.094	*p* = 0.256	*p* = 0.872
	iAG	8.0 ± 1.6	8.3 ± 1.7	η^2^_partial_ = 0.1	η^2^_partial_ = 0.1	η^2^_partial_ = 0.0	η^2^_partial_ = 0.1	η^2^_partial_ = 0.2	η^2^_partial_ = 0.1	η^2^_partial_ = 0.0

*Intramuscular lipid concentration%-value refers to the whole H_1_ signal.*

*BDC, baseline data collection; HDT59, day 59 of head-down tilt bed rest; CRTL, control group; cAG, continuous artificial gravity group; iAG, intermittent artificial gravity group.*

*Boldface indicates *P* < 0.05.*

The three-way mixed-model ANOVA on relative Δ revealed a main effect of *Muscle* (*p* = 0.035; η^2^_partial_ = 0.23) (Table 5). Pairwise comparisons Δ showed a greater increase in GMAX ILC than GMIN ILC (*p* = 0.038; CI 95%: 0.28–13.40). No main effect of *Group*, *Sex*, or interactions between factors were detected.

**TABLE 5:** Mean ± standard deviation of relative Δ of the gluteal muscle intramuscular lipid concentrations for the CTRL, cAG, and iAG groups.

**Group**	**Muscle (Δ)**			**Three-way mixed-model repeated-measures ANOVA**						
	**GMAX**	**GMED**	**GMIN**	**Muscle**	**Group**	**Sex**	**Muscle-by-group**	**Muscle-by-sex**	**Group-by-sex**	**Time-by-muscle-by-sex**
CRTL	7.0 ± 5.5	7.1 ± 13.7	−1.7 ± 5.0	*F*_2,26_ = 3.8	*F*_2,13_ = 0.4	*F*_1,13_ = 0.5	*F*_4,26_ = 0.8	*F*_2,26_ = 0.9	*F*_2,13_ = 0.7	*F*_4,26_ = 1.7
cAG	11.8 ± 6.1	7.0 ± 11.6	0.4 ± 7.3	***p* = 0.035**	*p* = 0.663	*p* = 0.490	*p* = 0.534	*p* = 0.403	*p* = 0.500	*p* = 0.173
iAG	7.1 ± 4.7	2.7 ± 5.6	4.6 ± 8.2	**η^2^_partial_ = 0.2**	η^2^_partial_ = 0.1	η^2^_partial_ = 0.0	η^2^_partial_ = 0.1	η^2^_partial_ = 0.1	η^2^_partial_ = 0.1	η^2^_partial_ = 0.2

*Intramuscular lipid concentration %-value refers to the whole H_1_ signal.*

*CRTL, control group; cAG, continuous artificial gravity group; iAG, intermittent artificial gravity group.*

*Boldface indicates *P* < 0.05.*

## Discussion

This study demonstrated that 60-day HDT bed rest induced muscle atrophy of all three gluteal muscles. However, ILC was only increased for the GMAX and GMED muscles, whereas no changes were detected for the GMIN muscle, which suggests differential accumulation of fat in the gluteal muscles after a prolonged period of decreased axial loading. This study also showed that daily AG did not mitigate the changes in muscle size and ILC.

### Gluteal Muscle Deconditioning During Bed Rest

The current results confirmed that prolonged HDT bed rest induced a reduction in the volume of all three gluteal muscles. The muscle volumes of the GMAX, GMED, and GMIN muscles were decreased by approximately 10, 8, and 11%, respectively. This decrease is similar to the 7% reduction in GMAX, calculated by averaging the results for the upper and lower volumes, a 4% reduction for GMED, and an 11% reduction for GMIN in a previous similar 60-day HDT bed rest study (Miokovic et al., 2011). The slight differences between studies might be explained by methodological differences, such as the use of different imaging equipment and slice thickness. The studies also varied with respect to the sex of the participants (Miokovic et al., 2011). Notably, the current study included female participants, unlike the previous study, which only included men (Miokovic et al., 2011). As males and females differ with respect to gluteal muscle size and activation, such as the GMAX muscle during running (Willson et al., 2012), the inclusion of females may explain some of the differences between the results of the two studies. Although no differences in the reduction in muscle volume were found between sexes in the current study, the current results need to be interpreted with caution due to the low number of female individuals and an unequal number of participants in the two groups. Future studies are recommended with a higher number of female participants.

There are some differences to other data that are accounted for by methodological factors. A shorter bed rest study (35-day HDT) with 10 male participants reported only a ∼2% decrease in the volume of the three gluteal muscles (Berg et al., 2007). In addition to the shorter exposure to bed rest, the imaging protocol also involved a single computerized tomography image acquired over the hip joint line. With respect to exposure to microgravity, another MRI study of four male astronauts after 17 days in space found that the GMAX muscle atrophied by ∼8% (Tesch et al., 2005). Despite the shorter duration of spaceflight, the reduction in the GMAX muscle volume was similar to the current results, which suggests that the deconditioning effects of microgravity in space might be faster than those of ground-based analogs (Hargens and Vico, 2016). Collectively, these results indicate atrophy of all three gluteal muscles during periods of decreased axial loading. A more standardized approach to data collection and data processing is necessary to compare changes in gluteal muscle size between space and ground-based studies.

A novel finding of the current study was the observed increase in the ILC in the GMAX and GMED muscles following 60-days of bed rest. Surprisingly, the GMIN muscle did not demonstrate any significant change in ILC, which suggests different adaptations of the individual gluteal muscles to decreased axial loading. Anatomical differences of the gluteal muscles might explain this finding. Compared with the GMIN muscle, the GMAX and GMED muscles are designed to bear a higher mechanical load, evident by their greater moment arms and greater physiological cross-sectional areas (Flack et al., 2012; Flack and Nicholson, 2014). During bed rest, these torque-producing muscles are in a relative state of disuse, as the load is eliminated in the axial direction. In contrast, the GMIN is argued to play a greater role in the control of the hip joint rather than torque production (Flack et al., 2012; Flack and Nicholson, 2014). On this basis, it might be expected that this muscle experiences a lesser reduction to its usual loading compared to the other gluteal muscles, and thus less increase in ILC during HDT bed rest. This differs from the pattern of ILC in gluteal muscles in adults with various hip disorders, including greater trochanteric pain syndrome (Ebert et al., 2019), hip fracture (Takano et al., 2018), and hip osteoarthritis (Zacharias et al., 2016). In these disorders, the ILC of the GMED and GMIN muscles is greater than that of healthy controls. The difference in the distribution of adaptation of the gluteal muscles between hip disorders and exposure to bed rest suggests differences in mechanisms. For instance, it is likely that joint conditions have a different impact on muscle loading. In joint pathology, muscle recruitment might be impacted by mechanisms such as reflex inhibition which does not necessarily unload the same muscles as HDT bed rest does. Painful conditions may also lead to disuse and deconditioning, whereas bed rest participants do not have underlying pathology contributing to the changes observed in their muscles. Further, other work highlights mechanisms unrelated to loading that might drive changes in muscle fat, such as inflammatory muscle responses, as has been shown for the paraspinal muscles in back pain (Hodges et al., 2014; James et al., 2018), and while these inflammatory changes are present with joint pathology, they might be unlikely with prolonged bed rest. Understanding the different increases in ILC in individual gluteal muscles may have implications for designing interventions to target the muscles with the greatest amount of deconditioning, which is important given that resistive exercise has recently been shown to mitigate the ILC of the trunk muscles (Welch et al., 2015), and that different exercises target individual gluteal muscles.

An additional consideration that may affect gluteal muscles in bed rest studies is the fluid shift out of the intramuscular space, both superiorly due to decreased axial loading on the circulatory system and laterally due to compression of the posterior hip on the bed. This phenomenon has been demonstrated within 120 min of bed rest (Greenleaf et al., 1977; Bosutti et al., 2020) and following spaceflight (Leach, 1979). Thus, the increase in ILC in the GMAX and GMED muscles may also partially reflect lower intramuscular water content rather than increased fat content.

### The Effect of Artificial Gravity on Gluteal Muscle Deconditioning

Contrary to the hypothesis of this study, 30 min of daily exposure to AG, either in a continuous bout or multiple intermittent bouts, was insufficient to mitigate gluteal muscle atrophy and increases in ILC. This lack of effect might be related to the limited duration of the exposure or the limited loading on the participants (1 Gz at the center of mass). During erect bipedal standing, the gluteal muscles have minimal myoelectric activity (Semciw et al., 2013, 2014). If the load on the hip muscles generated by AG in the current investigation was similar to that experienced by people standing on Earth, it is possible that the 30-min stimulus per day provided in the present study was insufficient to mitigate the changes in the gluteal muscles that are induced by disuse.

Alternatively, the absence of the effect of AG might relate to the neutral hip position of the participant while supine during the AG stimulus. Functionally, the GMAX muscle is mainly recruited to decelerate hip flexion from upright standing, such as during squatting, or to accelerate the body upward and forward from a hip flexion position, such as when climbing steps. The GMED and GMIN muscles mainly stabilize the hip joint and control pelvic drop during a single leg stance and gait, but minimal activity is recorded during a quiet bipedal stance (Gottschalk et al., 1989; Grimaldi et al., 2009). Thus, due to the supine, inactive position of the participants during centrifugation, the gluteal muscles were not stimulated in a sufficient manner to mitigate the deleterious effects of bed rest.

A previous study showed that, during 60-day HDT bed rest, including 3 days of 30-min resistive exercise per week prevented gluteal muscle atrophy compared with results from the control group (Miokovic et al., 2011). Several exercises were performed in a supine position, including bilateral squats (∼75–80% of pre-bed rest maximum voluntary contraction) and single-leg heel raises (∼1.3 times body weight) (Miokovic et al., 2011). Thus, for HDT bed rest studies in the future, a combination of bilateral squats and single-leg heel raises during AG in a supine position may provide a more effective countermeasure.

### Operation Relevance for Spaceflight

The NASA Human Research Roadmap highlights the importance of understanding human muscle physiology pre-, during and post-flight, and developing subsequent countermeasures against muscle deconditioning (Clément, 2017). With the current proposals regarding long-duration spaceflight, it is predicted that more people will experience spaceflight in the coming decades (Grenon and Saary, 2012). Preserving muscle function is a major priority for astronauts. In line with the known role of the gluteal muscles, the deconditioning of these muscles may negatively impact the function and control of the hip joint in astronauts. Therefore, the development of countermeasures is vital to ensure the wellbeing, mobility, and productivity of space travelers upon return to gravity fields. The present results highlight that these muscles are at risk for deconditioning but that the intervention trialed was ineffective at mitigating this.

### Further Directions

For future studies employing AG as an intervention, the daily duration of AG or the axial loading may need to be increased to mitigate gluteal muscle atrophy and increased ILC. However, increasing the load may be problematic, as some participants experience pre-syncopal symptoms even in 1 Gz (Frett et al., 2020). Alternatively, given that bilateral squats and single-leg heel raises have been shown to have a protective effect on gluteal muscle deconditioning associated with exposure to prolonged bed rest (Miokovic et al., 2011), combining the two countermeasures may provide a greater protective effect.

A more detailed spatial analysis of the gluteal muscles, which has previously been conducted for muscles of the cervical and lumbar spines (Abbott et al., 2015; Mhuiris et al., 2016), could provide crucial information regarding adaptations to decreased axial loading. For example, given that the upper and lower parts of the GMAX muscle have different biomechanical functions (Grimaldi et al., 2009), dividing the muscles in a supero-inferior manner may reveal the differential impact of bed rest across the different parts of the muscle. Additionally, increased ILC may be different in the anterior and posterior regions of the GMED and GMIN muscles. The anterior fibers of GMED and GMIN muscles facilitate hip flexion and internal rotation of the femur (Gottschalk et al., 1989), whereas the posterior fibers facilitate hip extension and external rotation (Gottschalk et al., 1989). An antero-posterior analysis may thus provide additional insight into the association between muscle deconditioning and hip function.

Although manual segmentation of muscles is a reliable way to measure volume and ILC (Smith et al., 2014), it is time-consuming. Using multi-atlas segmentation (Belzunce et al., 2020) or deep learning convolutional neural networks (Weber et al., 2019) to perform automatic measurement is more time-efficient and equally, if not more, reliable. These techniques could be used in future studies to allow advanced imaging technologies to monitor ILC changes, which may potentially be a new biomarker of muscle deconditioning.

### Limitations

The results of the current study should be interpreted while considering its limitations. First, the small sample size reduced the statistical power of the study and increased the likelihood of Type 2 errors, thereby missing any small differences between the three intervention groups. Because of the small sample size in the groups, only large effect sizes from countermeasures can be observed, and more subtle effects are likely to go unnoticed. However, it is difficult to increase participant numbers in these specialized studies, as bed rest facilities have limited capacity and are expensive to conduct. The current study is the first of multiple planned bed rest studies investigating passive AG and AG supplemented by exercises (Clément, 2017), and thus, the data from subsequent studies may be combined and will likely contribute to a future systematic review and meta-analysis. Another weakness of the sample is the uneven sex distribution (16 males, 8 females). The current study was the first of its kind to recruit female participants, and, as both sexes will travel into space or develop hip conditions on Earth, it is recommended that future bed rest studies include more females to allow further understanding of the sex differences in muscle atrophy and ILC.

Although bed rest is a useful analog for decreased axial loading, it only rotates the direction of gravity by 90° and does not eliminate gravity. During bed rest, participants most likely still recruited their gluteal muscles when performing daily activities, such as hygiene or bed transfers. Consequently, the decreased muscle volumes and increased ILCs may underestimate the true effect of microgravity. Future space studies are needed to investigate the translation of these findings.

The Dixon sequence was the chosen MRI technique as it reliably quantifies the amount of fat in an image. However, since both muscle and collagen appear dark in the fat-suppressed images, the gluteal muscle volume may be overestimated in the manual segmentation process (Reeder et al., 2016).

Finally, higher AG levels could mitigate muscle atrophy and increased ILCs in the gluteal muscles, but a higher rate of rotation may interfere with the physiological and psychological responses of the participants, as a load of >1G AG may cause discomfort and pre-syncope. Furthermore, whilst gravitational dose–response curves for some animal biochemical systems have been described (Wade, 2005), these dose–responses for most human physiological systems are unknown.

## Conclusion

This study demonstrated atrophy in all three gluteal muscles and an increased ILC in the GMAX and GMED muscles at the end of 60-day HDT bed rest. The AG protocols used in this study had no evident effects on the gluteal muscles. The demonstrated changes in the gluteal muscles are a potentially modifiable target for countermeasures against gluteal muscle deconditioning for astronaut and patient populations.

## Data Availability Statement

The original contributions presented in the study are included in the article/supplementary material, further inquiries can be directed to the corresponding author.

## Ethics Statement

The studies involving human participants were reviewed and approved by the Northern Rhine Medical Association (Düsseldorf, Germany, Application No. 2018143). The patients/participants provided their written informed consent to participate in this study.

## Author Contributions

NC, ED, DD, JS, TW, SS, PH, and JH: conceptualization and methodology. JZ: data collection. JE and MH: software. VT and ED: formal analysis and writing original draft preparation. VT, ED, JH, GC, JE, MH, JZ, KL, DD, AW, DB, JC, SS, TW, JS, PH, and NC: writing – review and editing. NC, AW, DB, and JC: funding acquisition. All authors have read and agreed to the published version of the manuscript.

## Conflict of Interest

TW and JS were employed by company KBR GmbH. The remaining authors declare that the research was conducted in the absence of any commercial or financial relationships that could be construed as a potential conflict of interest.

## Publisher’s Note

All claims expressed in this article are solely those of the authors and do not necessarily represent those of their affiliated organizations, or those of the publisher, the editors and the reviewers. Any product that may be evaluated in this article, or claim that may be made by its manufacturer, is not guaranteed or endorsed by the publisher.
